# Emerging trends in nucleic acid and peptide aptamers in plant science research

**DOI:** 10.1007/s00425-025-04637-w

**Published:** 2025-02-20

**Authors:** Kannath U. Sanjay, Chigateri M. Vinay, Navya B. Prabhu, Padmalatha S. Rai

**Affiliations:** Department of Biotechnology, Manipal School of Life Sciences, Manipal Academy of Higher Education, Manipal, 576104 India

**Keywords:** Nucleic acid aptamers, Aptasensors, Peptide aptamers, Bioimaging, Pathogen resistance, Functional genomics, Plant science

## Abstract

**Main conclusion:**

Aptamer technology has significantly advanced the field of plant research, emerging as a tool for enhancing agricultural productivity, plant growth, and environmental monitoring.

**Abstract:**

Aptamers are short nucleotide or amino acid sequences that can bind to a range of target molecules with high affinity and selectivity. In recent years, these affinity molecules have piqued the interest of researchers across various scientific fields, including pharmaceuticals, analytical chemistry, and plant science. Advancements in aptamer technology have significantly broadened the horizons of plant science, particularly in the areas of plant analyte detection, pathogen targeting, and protein function analysis. Despite the use of various other bioassays and molecular techniques for plant analyte detection, the small size, chemical stability, and cost-effective synthesis of aptamers make them invaluable tools for unravelling the complexities of plant cells. Here, we discuss the progress in the development of nucleic acid and peptide aptamers and summarize their applications in plant biotechnology. The principles and signalling methods of various aptamer-based biosensors and their prospects as biotechnological tools for functional genomic studies, pathogen resistance, and bioimaging are discussed. Finally, the present challenges and future perspectives of aptamer-based technology in plant research are also summarized.

## Introduction

The advancement of molecular tools has immensely transformed our understanding of plant biology and the intricate processes that contribute to plant development and growth. In recent years, aptamer technology has emerged as a vital tool with its exceptional applications in plant research. Aptamers are short, single-stranded nucleic acids or peptides that can bind to a range of targets, including proteins (Lautner et al. 2010; Abdeeva et al. 2019), phytohormones (Song et al. 2017; Sun et al. 2020), plant polysaccharides (Boese and Breaker 2007), small molecules (Mastronardi et al. 2021), as well as complex structures like cells and viruses (Ma et al. 2024). These highly specific affinity molecules remain distinct from traditional methods using antibodies and small molecular probes due to their reduced toxicity, immunogenicity, and ability to be synthesized on large scales without the use of animal hosts (Dunn et al. 2017; Allemailem et al. 2020). Over the past decade, aptamer research has thus gained considerable attention in a broad range of fields, including diagnostics, therapeutics, and drug delivery.

In plant research, nucleic acid (NA) aptamers find their use as recognition elements in biosensors for the detection of various plant analytes (Zhang et al. 2017; Komarova et al. 2018) and for the diagnosis of plant pathogens (Virk et al. 2024), while peptide aptamers are employed in functional genomic analyses, improvement of pathogen resistance (Bhor and Gosavi 2022), and plant breeding approaches (Gong et al. 2014a). Additionally, NA aptamers can be used for high-resolution bioimaging due to their ability to be integrated with organelle-directing or optical responsive groups (Khosravi et al. 2020; Głazowska and Mravec 2021; Mou et al. 2022), allowing targeted delivery of imaging agents to desired sites within a plant cell. This review aims to discuss the selection strategies, characteristics, and applications of nucleic acid and peptide aptamers in plant research. We also highlight the functionality and feasibility of different types of aptamer-based biosensors for detecting plant analytes. Furthermore, we discuss the challenges and future directions for unlocking their full potential in agricultural and plant sciences. For data collection, we searched bibliographic databases of scientific literature such as PubMed, Scopus, and Science Direct up to June 2024. All studies that reported the use of NA and peptide aptamers, specifically in plant samples and plant biotechnology, were included in the review.

## Aptamer selection strategies

Design strategies for NA and peptide aptamers aim to enhance their structural features, improving their binding affinity and stability under diverse physiological conditions. Both types of aptamers are typically identified through iterative selection processes that isolate high-affinity ligands from vast combinatorial libraries. While the following section outlines traditional selection methods, we note that advancements in computational technology and artificial intelligence (AI) have significantly transformed aptamer selection over the last decade. Various tools for high-throughput sequence analysis, 2D and 3D structure modelling, molecular docking, and molecular dynamics simulation are used to assess the stability of aptamer–target complexes and their binding energies (Poustforoosh et al. 2022; Fadeev et al. 2022; Sun et al. 2022). However, these tools are still not widely accepted due to certain limitations. Firstly, most approaches are laborious, computationally demanding, and require bioinformatics expertise. Secondly, the data utilized to train AI models are insufficient because only a few verified datasets are available in this area (Lee et al. 2023). Although they aid the selection process, computational techniques cannot replace experimental validation. A hybrid strategy that combines computational predictions with experimental validation is still needed to confirm the accuracy of the chosen aptamers.

## Nucleic acid aptamers

**Fig. 1:**
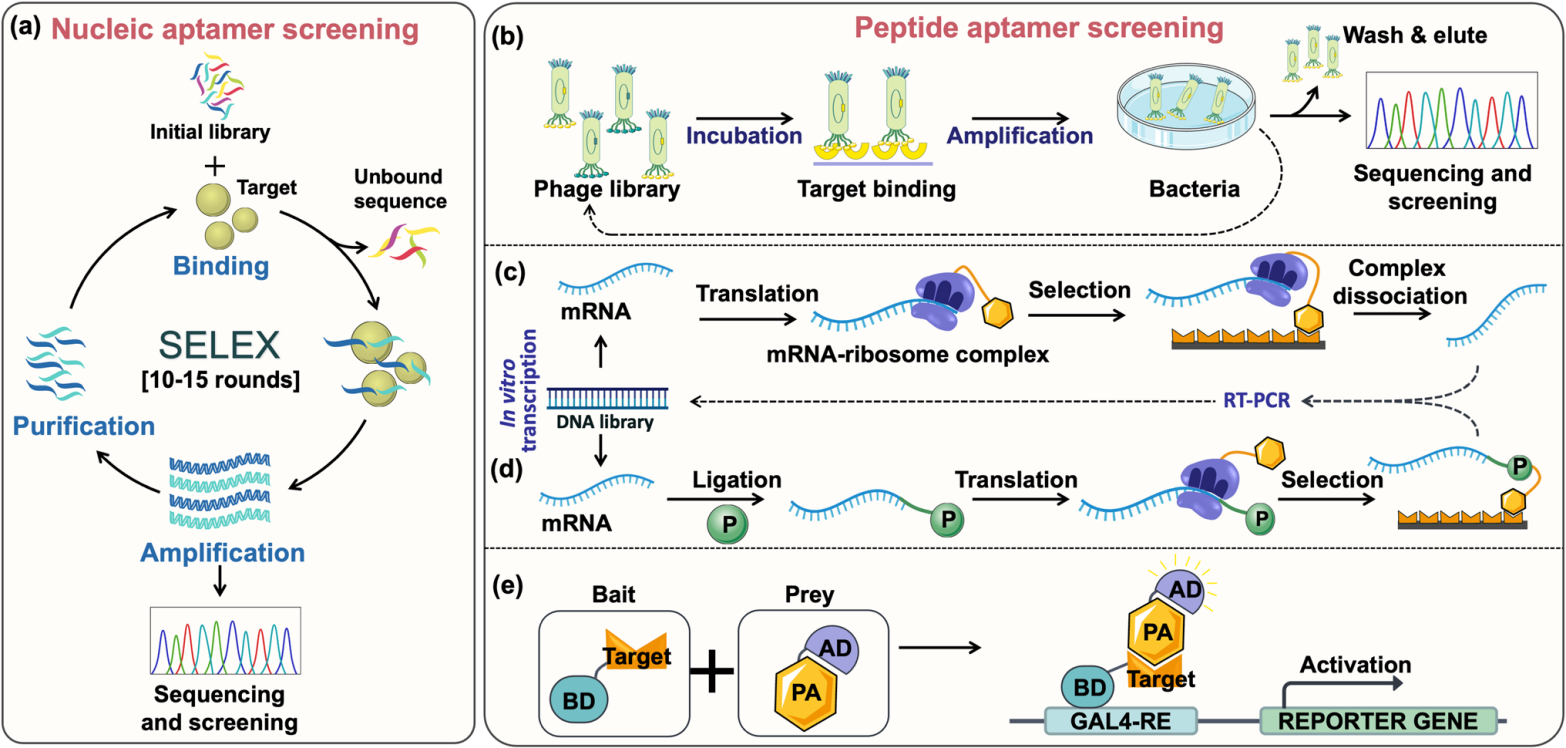
**a** Schematic representation of traditional SELEX for nucleic acid aptamer screening. Different strategies for peptide aptamer screening: **b** phage display library workflow, illustrating the method of biopanning for target binding, amplification of binders, and iterative selection to identify high-affinity peptides or proteins; **c** ribosome display workflow, showing in vitro transcription‒translation, formation of ribosome‒mRNA‒protein complexes, target binding, complex recovery, and iterative selection to identify high-affinity binders; **d** mRNA display workflow, depicting mRNA‒protein fusion via a puromycin (P) linker, target binding, complex recovery, and iterative selection to identify high-affinity binders; **e** yeast two-hybrid system, where a target protein (“bait”) is fused to a DNA-binding domain (BD) and a peptide library (“prey”) is fused to a transcriptional activation domain (AD). The interaction between bait and prey reconstitutes a functional transcription factor that activates a reporter gene, often detected via colorimetric assays (parts of the schematics are created with BioRender.com)

NA aptamers consist of single-stranded DNA, RNA, or Xeno nucleic acid (XNA) nucleotides (20–100) that bind with the target's specific site. The smaller size and ability to form compact 3D structures enable NA aptamers to create binding pockets or clefts for tight and specific binding. They interact with the targets of interest via electrostatic interactions, ℼ–ℼ stacking, hydrogen bonding, and van der Waals forces and can exist in various structural shapes (Cai et al. 2018). NA aptamers are selected in vitro from a random pool of oligonucleotide sequences by a combinatorial biology approach called SELEX (systematic evolution of ligands by exponential enrichment), discovered in the 1990s by two research groups led by Ellington and Tuerk (Tuerk and Gold 1990). SELEX technology primarily involves library preparation (10^13^–10^16^ sequences), followed by incubation with the target, separation of unbound sequences, and amplification of the bound sequences via polymerase chain reaction (PCR) (Fig. 1a). After multiple (10–15) rounds of screening, the selected aptamers are synthesized, and their binding affinity towards the target is investigated using via various biochemical and biophysical methods, such as surface plasmon resonance (SPR), isothermal titration calorimetry (ITC), or enzyme-linked oligonucleotide assay (ELONA) (Yüce et al. 2018). The conventional SELEX process is time-consuming and tedious and has a low success rate; methods such as additional washes, increasing salt concentrations, and subtractive capture to eliminate non-specific sequences are warranted after each round (Zhuo et al. 2017). Over the past few decades, advancements in SELEX-based techniques have made the selection of aptamers for small molecule targets more reliable and efficient (for a detailed review, see Zhang et al. 2019)).

These advanced SELEX techniques have been developed to optimize aptamer selection for different targets and applications. Capture-SELEX is effective for small solutes, leveraging hybridization to immobilize DNA libraries (Nutiu and Li 2005; Stoltenburg et al. 2012), whereas graphene oxide SELEX (GO-SELEX) avoids target immobilization by adsorbing ssDNA onto graphene oxide, making it suitable for certain applications (Lu et al. 2017). Capillary electrophoresis SELEX, or CE-SELEX, excels in selecting aptamers for protein targets by separating complexes in free solution via capillary electrophoresis, enabling rapid enrichment (Xiao et al. 2021). Each method addresses specific challenges, such as steric hindrance and false positives, or the need for target immobilization, highlighting the importance of tailoring the selection approach to the target and experimental context. These techniques have nevertheless been used in plant science for the efficient selection of various aptamers. For instance, Capture-SELEX was used by Komarova et al. (2018) to develop an ssDNA aptamer for furaneol, a flavour compound found in strawberries, raspberries, coffee, and wine, and by Zhang et al. (2017) to create an aptamer for geniposide, an iridoid glycoside from *Gardenia jasminoides fructus* (Zhang et al. 2017; Komarova et al. 2018). GO-SELEX was employed by Chergui et al. to generate a highly specific aptamer for glyphosate, a widely used herbicide. CE-SELEX has demonstrated its efficiency in protein targeting, as shown by Tran et al. (2013), who developed a DNA aptamer against Ara h1, a peanut allergen (Tran et al. 2013).

## Peptide aptamers

Peptide aptamers, also called affimers or peptimers, are short artificial peptides (8–30 amino acids) selected to specifically bind to the variable region of the target protein or peptide. They are displayed on an inert scaffold protein, such as thioredoxin A (TrxA) from *Escherichia coli,* or are embedded in a protein matrix as carriers. Further, when linked to an inert scaffold at both termini, termed constrained peptides, they exhibit more proteolytic stability and binding affinity than free peptide sequences (Reverdatto et al. 2015; Devi and Chaitanya 2022). The first peptide aptamer was selected through a combinatorial library approach targeting human cyclin-dependent kinase 2 by Colas et al. in 1996 (Colas et al. 1996). Although the technical features of peptide aptamer selection differ from those of NA aptamer selection, the fundamental steps remain the same. These strategies fall under the categories of non-display systems, cell-dependent display systems, and cell-free display systems. In non-display systems, the target protein is co-expressed in vivo alongside each sequence from the peptide. This approach eliminates the need for separate target protein display, relying instead on the host organism's ability to express the target protein in a correctly folded state. When a binder interacts with the target, it leads to the reconstitution of protein activity (e.g. transcriptional activity or fluorescence) in vivo (Mascini et al. 2012). Phage display is a cell-dependent selection system that uses bacteriophages as an expression vector. Briefly, the phage-replicating genome is modified to incorporate exogenous or foreign DNA, allowing the expression of the peptide of interest on the surface of the phage particle. Upon incubation with the target, the bound phages are eluted, grown in bacteria, and subjected to consecutive rounds of selection by biopanning (Fig. 1b). This method is widely used in aptamer selection due to the ease with which positive phages may be utilized to obtain all the genetic data required for peptide aptamer generation (Smith and Petrenko 1997; Malhis et al. 2021). However, library sizes can be limited by the efficiency of DNA transformation into cells, and the cellular toxicity of combinatorial library proteins remains a potential problem in the selection process.

Cell-free display systems, such as ribosome and mRNA display, allow the usage of protein libraries with 10^12^–10^14^ variants, in contrast to phage display and yeast two-hybrid (Y2H) systems, which are limited to 10^7^–10^9^ variants (Seelig 2011). In the ribosome display system, removing the stop codon from the template retains the mRNA–ribosome–peptide complex for target binding during selection. The resulting non-covalent complex formed between the mRNA, ribosome, and translated peptide is then utilized to bind to a fixed target during the selection phase. Post-selection, the complex is isolated, and the mRNA is used as a template for reverse transcriptase enzyme to retrieve the peptide aptamer sequence (Fig. 1c) (Hanes and Plückthun 1997). In the mRNA display system, a slightly modified library is used to yield translated peptides or proteins linked to their mRNA progenitor through puromycin linkage, as illustrated (Fig. 1d) (Roberts and Szostak 1997; Newton et al. 2020). The ideal selection technology for a specific protein library is determined by several factors, including library type and diversity, scaffold type, and its intended applications. The Y2H system is an in vivo selection technology in which a target protein is linked to the DNA binding domain (BD) of transcription factors, such as GAL4 and LexA, serving as a “bait” in a yeast test strain. The peptide library is linked to the transcriptional activation domain (AD), acting as the “prey”, as illustrated (Fig. 1e). When a peptide binds to a target protein, a functional transcription factor is formed, which subsequently activates the promoter of a reporter gene, which is often confirmed via colorimetric assays (Fields and Song 1989; Paiano et al. 2019; Shivhare et al. 2021). Y2H does not involve competitive binding during the selection process, allowing the simultaneous identification and selection of multiple peptide aptamers for the same target protein.

## Aptamer-based biosensors

Recent advancements in aptamer technology have enabled the creation of biosensors that can detect a range of plant analytes, including bioactive phytocompounds, phytohormones, pesticides, and biomarkers for crops and pathogens, instantly from plant cells, extracts, food products, and environmental samples (Wang et al. 2024). Aptamer-based biosensors, also known as aptasensors, utilize nucleic acid aptamers as recognition elements to detect specific target molecules. The high specificity, sensitivity, and non-toxicity of aptamers enable the detection of even low levels (nanomolar to picomolar range) of target analytes in complex biological systems like plant cells (Novák et al. 2017; Mou et al. 2022). Compared with other biosensing tools that may offer only a narrow range of detectable compounds, aptamers offer multiple advantages, such as ease of conjugation, immobilization, regeneration, and labelling, enabling us to develop numerous types of molecular probes (Kou et al. 2020; Li et al. 2021). Further, the feasibility of NA aptamers to easily undergo post-SELEX modifications makes them better recognition elements in biosensors, rendering them excellent candidates for point-of-care testing (Prante et al. 2020). They are categorized according to their signal transduction mode, including electrochemical, optical, and mass-sensitive sensors (Zahra et al. 2021). Optical aptasensors use various optical techniques to detect target molecules, including fluorescence, colorimetric, and surface-enhanced Raman spectroscopy (SERS)-based aptasensors, which have been reported for use in plant research. The conformational changes within the aptamer molecule driven by the target interaction are observed through the release of dyes, quenching of fluorescence, or other processes that optical instruments can detect.

**Fig. 2:**
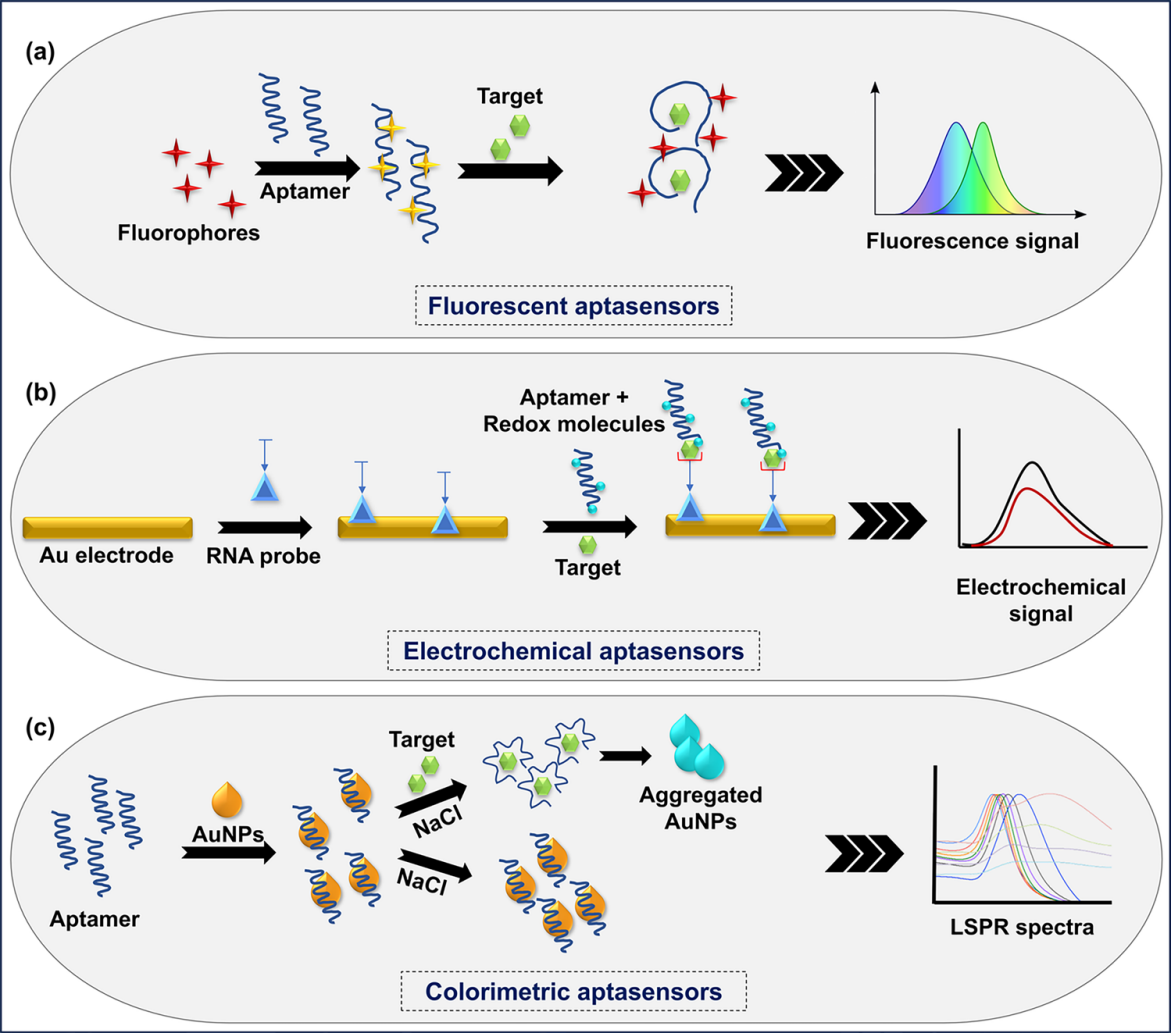
Working principle of commonly used aptasensors for detecting plant analytes: **a** fluorescent aptasensors, illustrating target binding to a specific aptamer, inducing a conformational change or displacement event that modulates the fluorescence signal for target detection; **b** electrochemical aptasensors, showing target binding to an aptamer immobilized on a gold (Au) electrode surface, leading to changes in electrochemical signals for target detection; and **c** colorimetric aptasensors, illustrating target binding to an aptamer, triggering a colour change in a substrate like gold nanoparticle (AuNP), which is used for visual detection of the target

Fluorometric aptasensors are a popular optical technique for developing rapid and sensitive biosensors utilizing fluorescent signalling/quenching caused by conformational changes (Fig. 2a) (Lee et al. 2021). These aptasensors operate through various mechanisms, including energy transfer, the G-quadruplex/probe complex, and competitive binding between complementary DNA (cDNA) and analytes (Zhou et al. 2020). They fall into two categories: labelled and label-free sensors. Labelled sensors are designed based on fluorophore molecules termed molecular beacons attached to the terminal ends of the aptamers. Molecular beacons are used for ribozyme activation and reading of aptamers (Le et al. 2014). Since this is a time-consuming, cost-ineffective, and laborious process that could alter the intrinsic binding affinity or specificity towards the target (Perez-Gonzalez et al. 2016), label-free sensors have been developed with the help of aptamer–complementary oligonucleotide (cDNA) duplexes and intercalating dyes as fluorescent probes for identifying analytes. In comparison, colorimetric aptasensors employ a more straightforward sensing strategy that monitors colour changes using UV–Vis spectroscopy or visual inspection by the human eye. A common approach involves the use of aptamers in conjugation with gold nanoparticles (AuNPs) and silver nanoparticles to develop these types of sensors. AuNPs, in particular, are favoured due to their unique properties, such as light scattering, fluorescence quenching, optical absorption, minimal toxicity, and a high extinction coefficient. Additionally, localized surface plasmon resonance (LSPR) is a distinctive feature of AuNPs that has been exploited in biosensing devices (Niu et al. 2014; Atapour et al. 2022). When aptamers are adsorbed on the surface of AuNPs, they stabilize the nanoparticles, preventing salt-induced aggregation due to the SPR effect. When a high-affinity target is added to the aptasensor, the aptamer binds to it, causing it to desorb from the surface of the AuNPs, resulting in aggregation of the AuNPs (Fig. 2c) (Chávez et al. 2010). In addition, optical aptasensors use the enhanced detection capacity of SERS, a powerful technique that significantly amplifies the Raman scattering signals of molecules adsorbed on rough metal surfaces or nanomaterials (Li et al. 2017). These aptasensors, where aptamers are immobilized on the surface of SERS-active substrates like gold or silver nanoparticles, rely on the internal harmonic vibration frequency and the vibration energy levels of molecules.

Electrochemical aptasensors have recently gained popularity because of their short response time, simplicity, portability, high sensitivity, and low cost (Hayat and Marty 2014). They are constructed by labelling the aptamer with redox molecules and immobilizing them on an electrode surface. The electrode surfaces are typically made of non-carbon-based materials (gold, silver, silicon oxide, and tin oxide) or carbon-based materials such as graphite and carbon nanotubes. The electrodes function as transducers, converting microcurrent or microvoltage into observable signals. When the aptamer binds to the target, the resulting charge flux causes variation in the electrochemical signals (Fig. 2b). These signals are recorded in the modes of potentiometry, electrochemical impedance spectroscopy, and voltammetry (Wang et al. 2020a; Villalonga et al. 2022). Various techniques are employed for the development of electrochemical aptasensors, such as target-induced displacement, turn-on and turn-off mechanisms induced by aptamer conformational changes, and nanoparticle-based signal amplification techniques (Trojanowicz 2014; Hosseinzadeh and Mazloum-Ardakani 2020). Thus, various types of aptasensors can be used for a wide array of plant analyte detection. Compared with conventional antibody-based biosensing tools, aptasensors are thermally stable, economical, rapid, and easily reproducible, making them more efficient and versatile in medicine, plant science, and food quality control.

## Nucleic acid aptamers in plant research

In plant research, NA aptamers have been applied in diverse fields, including environmental science (Hayat and Marty 2014; Nguyen et al. 2017; McConnell et al. 2020), agricultural biotechnology (Hu et al. 2022; Yang et al. 2023), and plant health monitoring (Tungsirisurp et al. 2023). Although mass spectrometry, genetically encoded biosensors, and immunoassays are commonly used to measure plant analytes, these methods require expensive, high-end instruments, specialized training, extensive sample pre-treatment, and are time-consuming. Another significant challenge in detecting and monitoring plant analytes is their low abundance, small size, and diversity, limiting our knowledge about the characterization and distribution of these molecules in plant systems (Novák et al. 2017). The development of aptamer-based biosensing and bioimaging tools aids in mitigating these challenges and expands our understanding of plant physiology and microenvironment. For instance, NA aptamers targeted against specific rhizosphere biomarkers are particularly interesting to researchers as they help determine the biological condition and state of soil and plants (Hu et al. 2022).

## Aptasensors for the detection of phytocompounds

The detection and quantification of plant analytes, including various bioactive compounds, phytohormones, and rhizosphere biomarkers, are crucial for understanding their role in plant biology, pharmacology, and environmental sciences. A comparison of different types of nucleic acid aptasensors used to detect plant analytes from plant samples is provided in Table 1. Theophylline, a xanthine derivative generally found in cocoa beans and tea leaves, was among the first bioactive compounds isolated from plants (Wrist et al. 2020). In 2018, a highly sensitive label-free aptasensor for theophylline detection was developed using nanopore structures (Feng et al. 2018). This cost-effective sensor could detect up to 0.05 µM theophylline from *Arabidopsis* plant extracts. Additionally, a recent study introduced an optical aptasensor system called the in situ plant metabolite aptasensor (IPMA), which is designed to detect and quantify methylxanthines, specifically theophylline and caffeine, directly from the leaves of *Ilex guayusa* (Intriago et al. 2022). Although this system presents the potential of theophylline detection in complex matrices like plant leaves, a significant drawback of this system is the high limit of detection (LOD), which must be overcome for practical use in plants. Studies have also shown the efficacy of electrochemical aptasensors for the sensitive detection of digoxin, a therapeutic compound extracted from *Digitalis purpurea*, from clinical samples (Bagheri et al. 2015; Wijesinghe et al. 2020). However, their use in detecting digoxin levels from plant samples has not been reported.

**Table 1:** Comparison of different types of nucleic acid aptasensors to detect plant analytes from plant extracts

Target molecule	Detection mode	Limit of detection (LOD)	Plant sample	References
Theophylline	Optical	50 nM	*Arabidopsis thaliana*	Feng et al. 2018
Berberine	Fluorescent	0.369 μg/mL	Kampo herbal formulations	Nuntawong et al. 2024
Abscisic acid	Colorimetric	330 nM	*Oryza sativa*	Wang et al. 2017
Abscisic acid	Colorimetric	100 nM	Plant extract	Song et al. 2017
Abscisic acid	Colorimetric	0.51 nM	*Oryza sativa*	Wang et al. 2020a, b
Abscisic acid	Fluorescent	30 ng/L	*Oryza sativa*	Shi et al. 2021
Abscisic acid	SERS	0.67 fM	*Triticum aestivum*	Zhang et al. 2022a
Abscisic acid	Fluorescent/SERRS	330 fM/ 38 fM	*Triticum aestivum*	Zhang et al. 2022b
Trans-zeatin	Electrochemical	16.6 pM	Plant extracts	Zhou et al. 2018
Trans-zeatin	Colorimetric	37 nM	Plant extracts	Sun et al. 2020
Salicylic acid	Optical	100 nM	*Arabidopsis thaliiana/Ozrya sativa*	Chen et al. 2019

Berberine, an isoquinoline alkaloid isolated from plants, is used to treat diabetes, skin diseases, infections, digestive disorders, and weight loss. The potential of berberine derivatives as light-up fluorescence probes has garnered interest due to their ability to specifically bind G-quadruplex (G4) structures and emit fluorescence upon binding, enabling the observation and analysis of G4s. DNA aptamers were selected for berberine using a modified affinity chromatography SELEX method. The selected 21-mer aptamer demonstrated greater fluorescence amplification, suggesting that the aptamer binds strongly and specifically to berberine, enhancing its fluorescent properties more effectively than G4 structures (Wang et al. 2016). The berberine–aptamer pair is a fluoromodule that can be incorporated into fluorescent sensors and labels for various detection purposes (Xu et al. 2015). Recently, a rapid fluorometric detection method for berberine was developed using colloidal gold nanoparticle-based SELEX (GOLD-SELEX) to isolate specific single-stranded DNA (ssDNA) sequences that can significantly enhance the fluorescence intensity of berberine. The assay effectively analysed 128 distinct medicinal formulations, exhibiting an LOD of 0.369 μg mL − 1 and remarkable selectivity in identifying berberine levels (Nuntawong et al. 2024).

Aptamer-based sensors are also powerful tools for detecting and monitoring phytohormones, crucial signalling molecules in plants that regulate growth, development, and responses to environmental stimuli. Abscisic acid (ABA) is a phytohormone for which a high-affinity aptamer was developed in 2013 by a direct SELEX method (Grozio et al. 2013). Wang et al. (2017) designed a low-cost and rapid aptasensor for detecting ABA from fresh leaves of rice based on the localized surface plasmon resonance (LSPR) of AuNPs. The hydrophobicity and molecular interactions of AuNPs with ssDNA were manipulated to synthesize a G-quadruplex DNA aptamer-based biosensor, which could be used for the detection of other plant analytes in addition to ABA (Wang et al. 2017). While this method was based on NaCl-induced AuNP aggregation with an LOD of 0.33 μM, a modified approach of polyadenine-tailed aptamer functionalization to AuNPs to construct a multi-channel apparatus yielded a lower detection limit of 0.51 nM from fresh leaves of rice (Wang et al. 2020b). The potential of combining advanced materials and innovative sensing techniques to achieve high sensitivity and selectivity in plant analyte detection was demonstrated by a study, in which a novel ratiometric fluorescent aptasensor was built by combining carbon quantum dots (CQDs), gold nanoparticles, and 2-methylimidazole zinc salt (ZIF-8) for ABA. Heavy-metal crystals, which produce fluorescence emission with high quantum efficiency, are the typical foundation for quantum dots. The highly sensitive ZIF-8-CQDs nanomaterial offered dual emission properties and stability, and this assay exhibited a detection limit of 30 ng/L (Shi et al. 2021).

Additionally, an SERS aptasensor was developed for detecting ABA from wheat (*Triticum aestivum*) leaves with a detection limit of 0.67 fM, which is significant compared with other existing aptasensors. They used magnetic ferric oxide nanoparticles as capture probes and silver-coated sulfhydryl-tagged aptamer (Au@AgNps-Apt) as signal probes. The signals are generated by the release of signal probes from capture probes in the presence of ABA, leading to variations in the SERS intensity (Zhang et al. 2022a). The same group developed another sensor utilizing the surface-enhanced Raman resonance scattering (SERRS) properties of gold nanorods and fluorescence quenching to detect ABA from natural wheat sample extracts. This SERRS and fluorescent dual-functional aptamer sensor exhibited a linear increase in the SERRS signal and fluorescence intensity as the ABA concentration increased from 1 × 10^−13^ to 1 × 10^−6^ M, with detection limit of 38 fM and 0.33 pM, respectively (Zhang et al. 2022b).

Trans-zeatin (Tz) is a significant cytokinin that regulates plant development and ageing by modulating cell division, tissue differentiation, and plant growth. A G4 DNA aptamer for Tz was selected by affinity chromatography SELEX with the highest binding affinity (Kd = 3.85 ± 0.05 μM) (Qi et al. 2013). An electrochemical aptasensor with an impressive LOD of 16.6 pM was developed for Tz using 2D layered molybdenum disulfide nanosheets, AuNPs, and an enzymatic signal amplification strategy (Zhou et al. 2018). Recently, AuNPs were used as colorimetric probe and structure-switching aptamer to detect zeatins from plant samples. The analytical results demonstrated a solid linear relationship from 0.05 to 0.75 μM and a detection limit of 0.037 μM (Sun et al. 2020). Salicylic acid (SA), another phytohormone that regulates immunological reactions against pathogens, is currently detected using costly and laborious methods. Chen et al. (2019) selected an aptamer with high affinity via the structure-switching SELEX method for SA. They developed a nanosensor that could even detect micromolar levels of SA from *Arabidopsis* and rice (*Oryza sativa*) extracts, which is much better than antibody-based approaches. These nanosensors have proven to be a promising sensing technique for identifying targets from plant extracts, buffers, and biological samples (Chen et al. 2019). In addition, highly specific aptamers have been selected against several rhizosphere biomarkers that can reflect the health and nutritional status of plants. For instance, DNA aptamers for the root exudate L-serine, a critical biomarker for nitrogen uptake in crops like wheat, have been identified (Mastronardi et al. 2021). However, their integration into functional aptasensors has not yet been achieved, highlighting the need for further development to translate these aptamers into biosensing tools for agricultural applications.

## Aptamer-based pathogen detection

**Fig. 3:**
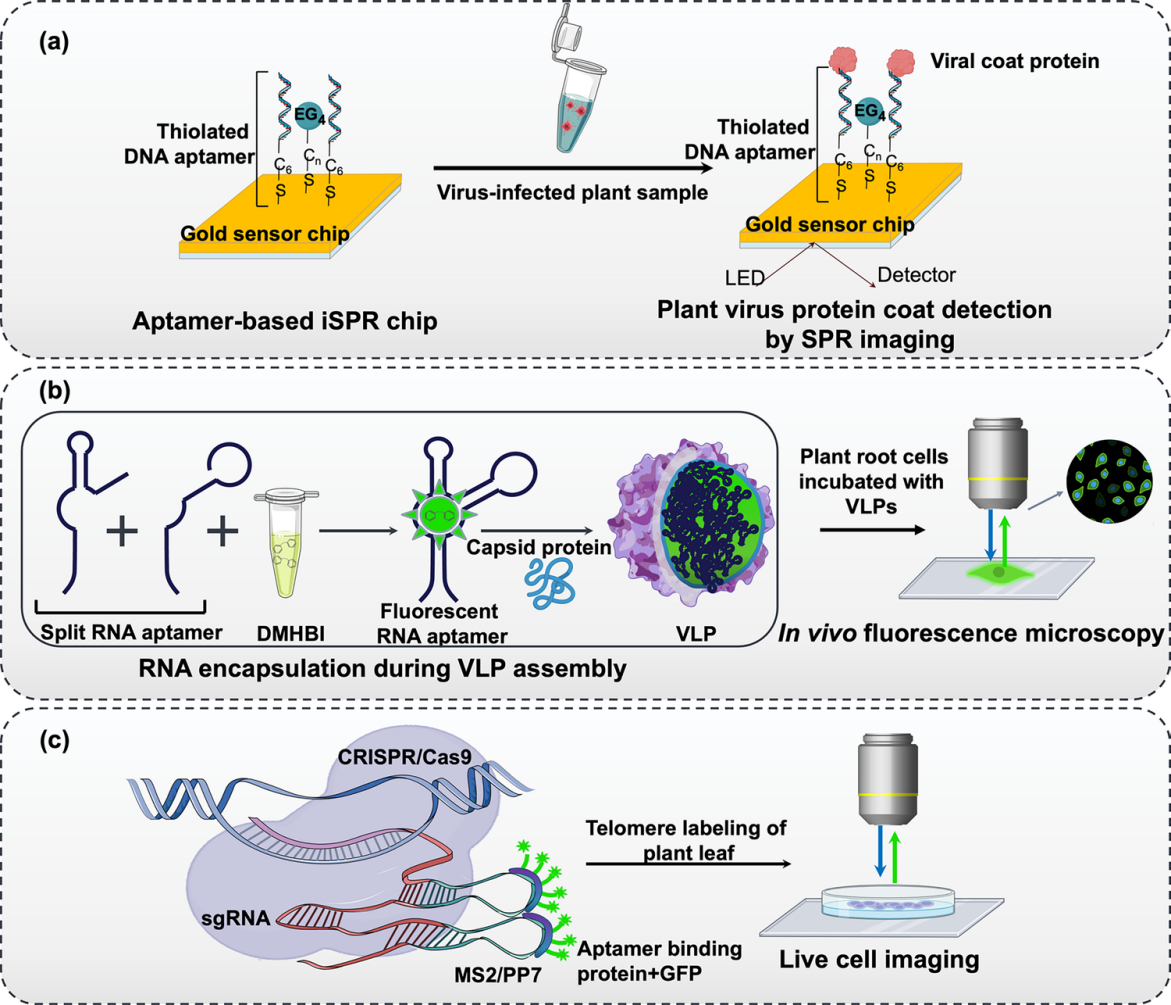
Schematic representation of aptamer-based pathogen detection and live-cell imaging in plants. **a** Surface plasmon resonance (SPR)-based aptasensor for detecting plant pathogens (e.g. virus particles). Thiol-labelled aptamers immobilized on a gold sensor chip via ethylene glycol (EG_4_) bind to target viral coat proteins, causing refractive index changes detected via SPR imaging (iSPR) for label-free detection (Lautner et al. 2010). **b** Segmented GFP-like aptamer probes for viral genome imaging. Aptamer components, along with fluorescent dye (DMHBI), assemble to form a functional probe, enabling fluorescence upon binding to the target viral RNA. The fluorescent signal is detected using microscopy, allowing visualization of viral genome trafficking in host cells (Tsvetkova et al. 2015). **c** Aptamers in CRISPR-based live imaging of plant telomeres. The sgRNA is engineered to include MS2/PP7 aptamer sequences, which bind to GFP-tagged aptamer-binding proteins, enabling fluorescent labelling of telomeres in plant leaves and facilitating live-cell imaging (Khosravi et al. 2020). DMHBI, 3,5-dimethoxy-4-hydroxybenzylidene imidazolone; VLP, virus-like particles; sgRNA, single guide RNA; GFP, green fluorescent protein (GFP) (parts of the schematics are created with BioRender.com)

Nucleic acid aptamers have also been used to detect plant pathogens, including viruses that pose a major threat to the agriculture industry. Two high-affinity aptamers against apple stem pitting virus (ASPV) coat proteins (sMT32 and PSA-H) with binding affinities (Kd) of 55 and 83 nM, respectively, were selected. Virus-infected plant extracts were identified using these aptamers by surface plasmon resonance (SPR) and dot blot. They also developed a double oligonucleotide sandwich enzyme-linked oligonucleotide assay (DOS-ELONA) for estimating coat protein concentrations. The sensitivity was higher than the commercial antibody ELISA kits (Balogh et al. 2010). SPR imaging (SPRi) is a label-free technique for real-time monitoring of biomolecular interactions and pathogen recognition (Kihm et al. 2012). Lautner et al. (2010) integrated SPRi and aptamer-modified sensor chips to detect apple stem pitting viruses from plant extracts (tobacco, apple or pear) by detecting the binding between the aptamer and the viral coat proteins, as illustrated (Fig. 3a) (Lautner et al. 2010).

## Nucleic acid aptamers to improve pathogen resistance

Various genetic engineering methods have been applied to create plants with enhanced pathogen resistance, especially agricultural crops. One promising avenue for developing plants with improved resistance appears to be the ability to bind specific proteins to an RNA aptamer and regulate their functions. Abdeeva et al. used an RNA aptamer to bind green fluorescent proteins (GFPs) and increase plant protein levels. Transgenic *Nicotiana benthamiana* plants expressing GFP showed a reduction in fluorescence when co-expressed with an RNA aptamer targeting GFP (Apt-Gfp) (Abdeeva et al. 2019). Another study by the same group targeted the extrinsic protein HopU1 of *Pseudomonas syringae*. Transgenic *Arabidopsis thaliana* plants expressing HopU1 aptamers exhibited disrupted HopU1 protein function and increased resistance to *P. syringae* (Abdeeva et al. 2023)(Table 2). These studies have demonstrated the application of RNA aptamers in enhancing pathogen resistance in plants (Abdeeva et al. 2024).

**Table 2:** Overview of aptamer technology for pathogen resistance in plants

Aptamer type	Target protein/enzyme	Target pathogen	Plant sample	References
Nucleic acid	HopU1 protein	*Pseudomonas syringae*	*Arabidopsis thaliana*	Abdeeva et al. 2023
Peptide	–	*Colletotrichum gloeosporioides*	*Stylosanthes sp.* (pencilflower)	Xu et al. 2022
	Cellulase synthase 2	*Plasmopara viticola*	*Vitis vinfera* (grape)	Colombo et al. 2020
	AL1 Replication initiator protein	*Gemiviruses sp.*	*Solanum lycopersicum* (tomato)	Reyes et al. 2013
	AL1 Replication initiator protein	Tobacco golden mosaic virus	*Solanum lycopersicum* (tomato)	Lopez-Ochoa et al. 2006
	Nucleocapsid protein	Tomato spotted wilt virus	*Nicotiana benthamiana* (tobacco)	Rudolph et al. 2003

## Aptamer-based applications in live-cell bioimaging

Bioimaging in plants can leverage aptamers as molecular probes or contrast agents to monitor the dynamics and distribution of molecules. By modifying aptamers with organic dyes, fluorescent dyes, radioisotopes, or nanoparticles, specific targets in higher plants can be visualized, providing valuable insights into the intricacies of plant systems (Fu et al. 2020). Their ease of conjugation with multiple imaging agents has been exploited in diverse modalities, such as magnetic resonance imaging (MRI), computed tomography (CT), SPRi, nuclear imaging, and ultrasound imaging. A notable example includes the study of Tsvetkova and colleagues, who developed a split RNA aptamer capable of encapsulating virus-like particles (VLPs) and functioning similarly to GFPs. This system was used to image the co-packaging of viral genomes during virion assembly and trafficking of Brome mosaic virus (BMV), an RNA virus that infects cereal plants. The fluorescence of the aptamer‒ligand complex varied based on the presence of RNA during VLP assembly, which limited the fluorophore's access to the aptamer or altered the ligand-binding site. Notably, increased fluorescence emission was observed inside the cell area closer to the cell wall, demonstrating that VLPs could penetrate the cells of live barley root cells and allow the study of intracellular processes in real time, as illustrated (Fig. 3b) (Tsvetkova et al. 2015).

Genetically encoded RNA probing systems such as MS2, boxB/λN22, CRISPR-Cas, and PP7 have been used as recognition elements in sensors that emit fluorescence upon binding to their targets in living cells. Over the past few years, researchers have tailored this system with various regulatory proteins, promoters, and aptamers for various purposes, including epigenome editing, genetic modifications, the regulation of chromatin topology, and live chromatin imaging (Zhang et al. 2016; Sun and Zou 2022). For example, aptamers have been employed in CRISPR/Cas9 systems to regulate targeted gene expression by attaching effector proteins, such as transcription activation domains, acetyltransferases, or methyltransferases, to aptamer-binding proteins (Selma et al. 2019; Lee et al. 2019). The effectiveness of the MS2–CRISPR/dCas9 system in modulating the transcriptional activity and epigenetic status of specific target genes in plants has been demonstrated in a study to achieve altered flowering time phenotypes after transformation into *A. thaliana* (Lee et al. 2019). This allows for precise control of gene activity by recruiting specific functional proteins to precise locations within the genome. Dreissig and colleagues established a method for live-cell imaging to visualize telomere repeats in *N. benthamiana* leaf cells using two orthologues of CRISPR–Cas9 fused with the fluorescent protein eGFP/mRuby2 (Dreissig et al. 2017). This approach allows for tracking dynamic telomere movements up to 2 μm over 30 min during interphase and has the potential to visualize multiple genomic loci and DNA‒protein interactions simultaneously. An advancement in this approach involves an aptamer-based chromatin imaging strategy comprising a 3-component labelling system that includes a catalytically inactive dCas9 regulated by the ubiquitin parsley promoter and a modified sgRNA with an aptamer sequence (PP7 or MS2) fused to fluorescent proteins (Fig. 3c). These components are directly recruited to the target molecule, enabling precise visualization with minimal background labelling noise and significant improvement in telomere labelling efficiency in comparison to other dCas9:GFP systems. The strategy was successful in transiently transforming *N. benthamiana*; however, they could not apply the same to stably transformed plants (Khosravi et al. 2020). Nonetheless, this aptamer-based strategy provides a new method for studying chromatin dynamics and gene regulation in plant cells, with potential applications in plant breeding and genetic engineering.

## Applications of peptide aptamers

Peptide aptamers regulate metabolic processes, phenotype changes, and protein functions in vivo and are also used as biologically active agents to inhibit the protein functions of phytopathogens. They have been used in bacterial, yeast, mammalian, and plant systems to obstruct protein activity. They can be employed in genetic investigations as “pertubagens” to locate the biochemical components and processes linked to particular cellular phenotypes (Crawford et al. 2003; Hoppe-Seyler et al. 2004; Yuan et al. 2024).

## Functional investigations of plant proteins using peptide aptamer-based technologies

Compared to NA aptamers that exhibit specificity towards a diverse array of biomolecules, peptide aptamer research is focused on the selective binding of proteins (Hao et al. 2018). Owing to their ability to inactivate a target protein under intracellular conditions, they are used for functional protein analysis. For instance, a 16-amino acid peptide aptamer (PAP) was developed via Y2H assay by Gong et al.(2014a, b) to specifically target MAGO proteins in rice (*Oryza sativa*), which are an essential part of the exon junction complex (EJC) involved in post-transcriptional processes, growth, development, and adaptive evolution in plants. Additionally, PAP transgenic rice plants had phenotypic defects that were comparable to those seen in rice MAGO and MAGO-Y14 RNAi lines, demonstrating that the competitive binding of PAP to MAGO can impair the natural MAGO-Y14 heterodimer's ability to function. Compared to the PAP-GFP chimaera, PAP demonstrated superior expression and a stronger affinity for MAGO proteins, suggesting that fusion with a protein scaffold may not be necessary for utilizing aptamers in higher plants (Gong et al. 2014b, a). In 2013, Song and colleagues screened a range of constructs with alanine substitutions for every individual amino acid against the coding region of the CLAVATA 3 (CLV3) gene, which is a glycosylated peptide hormone that regulates the number of stem cells in shoot apical meristems. These constructs were expressed and successfully transformed into *Arabidopsis,* revealing a conserved glycine residue present in the coding region of the CLE motif that caused a dominant-negative phenotype like that of CLV3. Furthermore, they reported that substituting glycine with threonine resulted in a more robust dominant phenotype (Song et al. 2013).

## Peptide aptamers to improve resistance against pathogens

A diverse range of pathogens, such as viruses, fungi, nematodes, and bacteria, can impact plants. Resistance to pathogens is vital for ensuring food safety and reducing reliance on chemical pesticides by promoting natural resistance. Since no antiviral chemicals are available to protect crops or plants, techniques such as crop rotation and integrated vector management are employed. However, certain limitations hinder their effectiveness, including the development of resistance and time-consuming breeding processes. Nucleic acid-based methods like RNA interference constructs, antisense RNAs, and CRISPR–Cas systems offer control, but are only effective against specific or closely related viruses (Shepherd et al. 2009). Using peptide aptamers for viral targets can provide high specificity and obstruct viral assembly or replication by producing a dominant interfering peptide, thereby creating broad-spectrum virus resistance in plants. Table 2 provides an overview of the peptide and NA aptamers used for pathogen resistance.

One of the pioneering studies in this regard reported the selection of a 29 aa peptide aptamer fused to a β-glucuronidase (GUS) scaffold protein against the tomato spotted wilt virus (TSWV) nucleocapsid protein using the Y2H system. The developed transgenic *N. benthamiana* plants overexpressing the 29 aa aptamer were resistant to four different tospoviruses, namely, TSWV, tomato chlorotic spot virus (TCSV), groundnut ring spot virus (GRSV), and chrysanthemum stem necrosis virus (CSNV), but none showed resistance to Impatiens necrotic spot virus (INSV) (Rudolph et al. 2003). In a subsequent study targeting geminiviruses, peptide aptamers were developed to interfere with the function of the highly conserved protein AL1/Rep, crucial for viral replication in plant cells. The study discovered 31 peptide aptamers using a Y2H screening that considerably decreased the replication of tomato golden mosaic virus (TGMV) DNA in tobacco protoplasts, resulting in a 13–64% reduction in viral DNA levels when compared to wild type (Lopez-Ochoa et al. 2006). Further research revealed that two of these aptamers, A22 and A64, strongly interacted with the terminal region of the Rep/AL1 protein in nine different geminiviruses. When these aptamers were expressed in transgenic tomato plants, they exhibited significant resistance, showing delayed or reduced symptoms and lower viral DNA levels upon infection with tomato yellow leaf curl virus and tomato mottle virus (Reyes et al. 2013).

Recent developments in employing aptamer technology for pathogen resistance include the development of an antimicrobial peptide selected against *Plasmopara viticola* Berl using the Y2H approach with *P. viticola* cellulose synthase 2 as bait. *P. viticola* causes downy mildew in grapevine (*Vitis vinifera*), resulting in substantial economic losses. The 8 aa aptamer, termed NoPv1, effectively prevents *P. viticola* germ tube formation and leaf infection while being safe for non-target organisms and human health. The aptamer also exhibits potential against *Phytophthora infestans,* likely due to similarities in cellulose synthase enzymes (Colombo et al. 2020). Additionally, Xu et al. (2022) recently reported the efficacy of peptide aptamers as a sustainable and potent antifungal treatment against the pathogenic *Colletotrichum gloeosporioides* on *Stylosanthes* plants. The construction of a nanocomposite consisting of a peptide aptamer enclosed within montmorillonite enhanced the aptamer's adhesion to leaves, resulting in comparable efficacy to commercial fungicides against anthracnose on *Stylosanthes* leaves, even at low concentrations (Xu et al. 2022). Overall, the applications of peptide aptamers described here may facilitate the development of long-lasting crop resistance, a goal that is challenging to achieve with conventional methods.

## Conclusions and future prospects

In the last 25 years, aptamers have facilitated the development of bioanalytical sensing tools, bioimaging tools and targeted delivery agents. NA and peptide aptamers are valuable tools for addressing various practical issues that require robust and selective binding to a particular target. This review synthesizes findings from various studies on the diverse applications of aptamer technology in plant research. Notably, their use as aptasensors for the detection of various plant analytes, including rhizosphere biomarkers (Mastronardi et al. 2021), phytohormones (Chen et al. 2019; Zhang et al. 2022b), and other bioactive compounds (Feng et al. 2018), has been reported. Aptamers also offer significant benefits in recognizing specific pathogen strains and enhancing resistance (Komorowska et al. 2017; Abdeeva et al. 2023), owing to their ability to be fine-tuned for specificity through advanced selection techniques. Additionally, peptide aptamers can interact with specific plant proteins and guide gene editing tools such as CRISPR/Cas, influencing plant physiological processes without altering plant genotypes or disrupting gene/protein structure. This allows for precise genetic modifications and the development of desirable attributes such as resistance to environmental stress or improving growth characteristics. Transgenic plants with constitutive transcription of highly effective aptamers to inhibit effector proteins are produced and employed in agriculture with the aid of contemporary genetic engineering approaches. In addition to traditional plant genetic transformation, peptide aptamers can be delivered using artificial synthesis, root-zone irrigation, and cell-penetrating peptide (CPP)-mediated techniques (Eggenberger et al. 2011; Colombo et al. 2015). However, discussing the practical efficacy of these technologies is challenging and requires further validation of their phytotoxicity, side effects, and practical implications in the field.

Notably, the potential applications of aptamers in plant research extend well beyond current uses, offering substantial opportunities for future development and innovation. While aptamer technology combined with advanced imaging techniques like super-resolution microscopy and single-molecule imaging has already been utilized in plant science (Głazowska and Mravec 2021), insights from other fields can further expand its applications. For example, covalent self-caging of aptamers for temporal control of biosensor activation, as shown by Tan et al. (2016), dramatically improves the binding of aptamer aptamers even at high concentrations of the target molecule (Tan et al. 2016). Additionally, organelle-specific photoactivation of DNA nanosensors (Shao et al. 2021) could be adapted to plants for spatially resolved analysis of enzymatic activities within specific organelles. This combination can provide high-resolution insights into plant biological pathways and regulatory networks and offer exceptional clarity and precision in visualizing molecular interactions and structures at the nanoscale level. Moreover, the challenges associated with delivering engineered nanomaterials (ENMs) or imaging agents to plants, such as the difficulty of crossing the plant cell wall, limited delivery efficiency, and the ability to target specific molecules, can be effectively addressed using aptamers as delivery vehicles (Mou et al. 2022). By enhancing targeted delivery, these ENMs can optimize agricultural methods and address problems with both traditional and contemporary farming practices involving agrochemicals. These strategies could pave the way to effectively control plant diseases and detect biomarkers and pathogens. Additionally, a novel approach of integrating a theophylline RNA aptamer to a DNAzymes (a DNA-based computing device) has been demonstrated to detect theophylline in clinical settings (Harding et al. 2022). This innovative method holds potential for application in biosensing platforms for plants, enabling advanced monitoring and detection of specific compounds. However, aptamers are still in the early stages of development in plant science and warrant intensive and focused research to extend their applications completely. While aptamers offer advantages over antibodies, comparing nucleic acid and peptide aptamers is challenging, as it involves evaluating various factors, including their binding affinity, synthesis complexity, and biological applicability. The choice between these types thus depends on the specific requirements of the application, such as the need for in vivo stability or the ability to interact with complex biological systems. Furthermore, the aptamer's characteristics and functionalities can be enhanced via chemical alterations and sequence changes (Zhao et al. 2022; Cai et al. 2023). Improvements in the development of aptasensors, including portability, digitization, simplification, and numerous factors like the complexity of plant matrix compounds, pH and temperature that may interfere with aptamer-target interactions, must also be considered to transform the already established aptamers into functional, practical point-of-care devices.

In summary, aptamers have proven to be a multifaceted tool in agriculture and plant research. The potential of this technology is in demand not only for the creation of sensors and test systems, but also for the search for alternative pesticides and biologically active substances, the creation of delivery systems, etc. The aptamer-based approach to identifying bioactive compounds corresponds to a significant advancement in the field, especially given the complex nature of herbal formulations. The rapid detection time of this assay presents a notable advantage, particularly in the herbal medicine industry (Nuntawong et al. 2024). These approaches open new avenues for exploring plant physiology and pathology with unprecedented detail, significantly advancing our understanding of complex plant systems. We believe that in the coming decades, in the context of the growing importance of plant-based products, the use of aptamers in this area will grow.

## Data Availability

No datasets were generated or analysed during the study.

## Abbreviations

NA: Nucleic acid
XNA: Xeno nucleic acid
ITC: Isothermal titration calorimetry
SELEX: Systematic evolution of ligands by exponential enrichment
TrxA: Thioredoxin A
SNase: Staphylococcal nuclease
BD: Binding domain
AD: Activation domain
GFP: Green fluorescent protein
AI: Artificial intelligence
Y2H: Yeast two-hybrid system
TAD: Transcriptional activation domain
SERS: Surface-enhanced Raman spectroscopy
AuNPs: Gold nanoparticles
SPR: Surface plasmon resonance
Kd: Dissociation constant
ABA: Abscisic acid
LOD: Limit of detection
LSPR: Localized surface plasmon resonance
CQDs: Carbon quantum dots
ZIF-8: Methylimidazole zinc salt
SERRS: Surface-enhanced Raman resonance scattering
Tz: Trans zeatin
SA: Salicylic acid
ASPV: Apple stem pitting virus
DOS-ELONA: Double oligonucleotide sandwich-enzyme-linked oligo-nucleotide assay
SPRi: SPR imaging
MRI: Magnetic resonance imaging
CT: Computed tomography
EJC: Exon junction complex
TCSV: Tomato chlorotic spot virus
GRSV: Groundnut ring spot virus
CSNV: Chrysanthemum stem necrosis virus
INSV: Impatiens necrotic spot virus
TGMV: Tobacco golden mosaic virus
ENM: Engineered nanomaterial
CPP: Cell-penetrating peptide
IPMA: In situ Plant Metabolite Aptasensor
